# Sclerosing peritonitis presenting as complete mechanical bowel obstruction: a case report

**DOI:** 10.1186/s13256-019-2243-0

**Published:** 2019-10-17

**Authors:** Sabah Uddin Saqib, Inam Pal

**Affiliations:** Department of General surgery, Aga Khan University Hospital, Karachi, Pakistan

**Keywords:** Complete mechanical bowel obstruction, Sclerosing peritonitis, Abdominal cocoon syndrome

## Abstract

**Introduction:**

Sclerosing peritonitis or abdominal cocoon syndrome is characterized by small bowel loops completely encapsulated by a fibrocollagenous membrane in the center of the abdomen. Although cocooning of the abdomen is mostly seen in patients on peritoneal dialysis, it can occur *de novo*; it very rarely manifests as complete mechanical bowel obstruction.

**Case presentation:**

A 46-year-old Asian man presented with complete mechanical bowel obstruction. He had previous attacks of partial bowel obstruction during the past 6 to 8 months, which was misdiagnosed as abdominal tuberculosis because tuberculosis is very prevalent in the region in which he lives. He took anti-tuberculosis therapy for 3 months but this did not result in resolution of his symptoms. This time he had diagnostic laparoscopy followed by laparotomy in which a fibrocollagenous membrane, resulting in entrapment of his bowel, was excised and his entire small bowel was freed.

Postoperatively he again had a mild episode of partial bowel obstruction but this was relieved with a short course of steroids.

**Discussion:**

Sclerosing peritonitis is a rare benign etiology of complete mechanical bowel obstruction. Patients might have suffered recurrent attacks of partial bowel obstruction in the past that were falsely managed on lines of other conditions such as tuberculosis, especially in endemic areas like Pakistan or India.

**Conclusion:**

Sclerosing peritonitis is a rare benign diagnosis which can manifest as complete bowel obstruction and a high index of suspicion is required to diagnose it. Contrast-enhanced computed tomography of the abdomen is a useful radiological tool to aid in preoperative diagnosis. Diagnostic laparoscopy is usually confirmatory.

Peritoneal sac excision and adhesiolysis is the treatment and a short course of steroids in relapsing symptoms.

## Introduction

Abdominal cocoon syndrome or sclerosing peritonitis is a rare condition that refers to total or partial encapsulation of the small bowel by a fibrocollagenous membrane resulting in partial or complete mechanical bowel obstruction [1]. The condition is mostly seen in patients with end-stage renal failure requiring peritoneal dialysis (PD) but it can occur without any pre-existing risk factor [2]. We report a case of an adult patient who presented with features of acute intestinal obstruction on a background of intermittent attacks of partial bowel obstruction for the past 6 to 8 months. He was treated surgically followed by a short course of steroids for his relapsing attack of partial bowel obstruction.

## Case presentation

A 46-year-old Asian man presented with 5-day history of absolute constipation, vomiting, and central abdominal pain. Besides that he had no known comorbidities and an unremarkable family history. In the past he had similar complaints for which he was managed conservatively. At 4 months prior to his presentation he had a computed tomography (CT) scan of his abdomen for similar symptoms; the CT scan showed ileal thickening for which he was given empirically a 3-month course of anti-tuberculosis therapy (ATT) but his symptoms did not resolve.

On this occasion, an examination revealed a dehydrated patient with pulse of 104 beats/minute and blood pressure (BP) of 130/70 mm. He had abdominal distention and central abdomen tenderness and hyperactive gut sounds. A digital rectal examination was unremarkable and so was a systemic examination. His baseline workup showed blood urea nitrogen of 32 mg/dl and creatinine of 1.2 mg/dl.

**Fig. 1:**
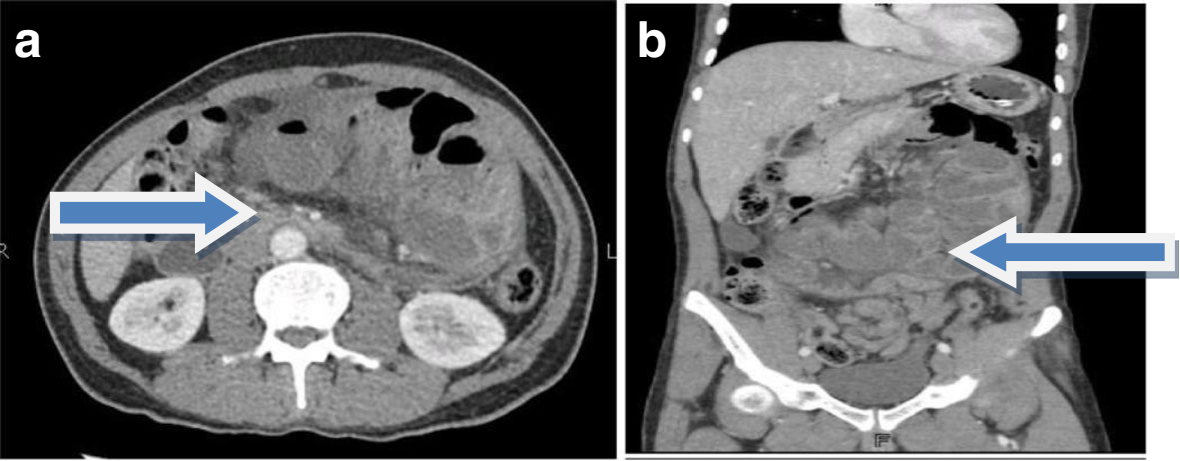
**a**, **b** Axial and coronal views of contrast-enhanced computed tomography demonstrates cocooning of abdomen shown by arrows

Contrast-enhanced CT (CECT) of his abdomen showed mildly dilated thickened jejunal and ileal loops which were encased in a thick fibrocollagenous membrane pushed in the center of his abdominal cavity with collapsed loops of large bowel; the findings were suggestive of sclerosing encapsulating peritonitis/abdominal cocoon (Fig. 1 a, b).

**Fig. 2:**
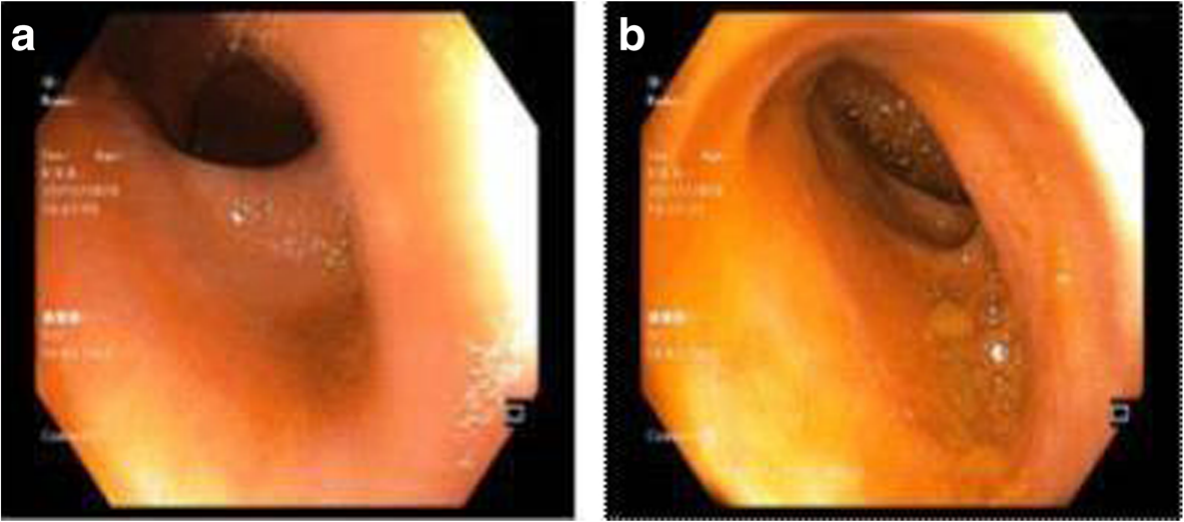
**a**, **b** Normal terminal ileum on colonoscopy

He was initially managed conservatively with intravenously administered fluids and nasogastric tube which resulted in some relief of his symptoms and his pulse of 74 beats/minute. Because of the fact that he came from an area where tuberculosis (TB) is a highly prevalent disease and previously he was empirically treated for abdominal TB, he underwent colonoscopy which showed normal terminal ileum, colon and rectum (Fig. 2 a, b).

His case was discussed in a multidisciplinary team, which included a radiologist, gastroenterologist, and gastroenterology surgeon, and he was planned for diagnostic laparoscopy, followed by laparotomy in case it was not abdominal TB or a malignancy requiring medical management only.

**Fig. 3:**
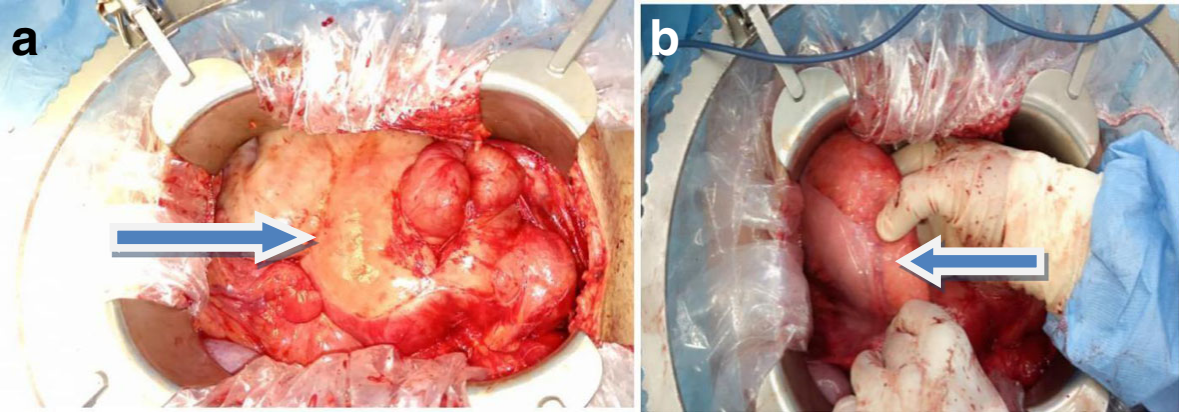
**a** Whole small bowel is encapsulated in a membrane, shown by arrow. **b** Thick stomach, shown by arrow

A diagnostic laparoscopy using 10 mm infraumbilical port in a vertical fashion, confirmed that entire small bowel was encapsulated in membrane and it was all plastered in the center of his abdomen. Hence, a decision was made for midline laparotomy, in which thickened sclerosing membrane encapsulating loops of small bowel was removed and whole small bowel was freed and run until ileocecal junction. His stomach appeared thickened while his colon appeared grossly unremarkable (Fig. 3 a, b).

Postoperatively he remained well and was discharged on fourth postoperative day, when he was tolerating an oral soft diet. However, he was again admitted on third day after his discharge with complaints of vomiting and relative constipation. He was kept nil by mouth (NPO) and on parenteral nutrition. Along with conservative management he had a short course of hydrocortisone 50 mg thrice daily for 7 days, which was tapered off later; he responded very well and he was discharged in a stable condition on oral soft diet with normal bowel movements.

He was followed up in clinic after 10 days and he was tolerating a soft diet with normal bowel movements; his stitches were removed in clinic. Later, a histopathology report showed fibrocollagenous tissue with mild chronic inflammation and mild patchy increase in IgG4-positive plasma cells.

He was seen twice as an out-patient at 3-month intervals and appeared asymptomatic; he was advised to have further follow-up only if required.

## Discussion

Sclerosing encapsulating peritonitis was first described more than a century ago and was initially termed peritonitis chronica fibrosa incapsulata to describe the membrane encasing the intestine; it has since also been named ‘icing sugar’, fibroplastic peritonitis, and cocoon abdomen. Sclerosing encapsulating peritonitis has been classified as primary and secondary based on whether it is idiopathic or has a definite cause.

The etiology of the primary form is uncertain with various hypothesis, although it is probably caused by a subclinical peritonitis leading to the formation of a cocoon [3–5]. Cytokines and fibroblasts probably influence the development of peritoneal fibrosis and neoangiogenesis in some way [6].

Secondary sclerosing peritonitis, which is more common, has many causes. The predominant cause of sclerosing peritonitis is PD. Patients on PD are predisposed to developing peritoneal deterioration after prolonged exposure to PD fluids and subsequent bacterial peritonitis [7, 8]. Other known causes include: recurrent peritonitis; abdominal TB; autoimmune diseases such as systemic lupus erythematosus, peritoneal shunts, and sarcoidosis; and ovarian disorders such as rupture of dermoid cyst.

The results of baseline investigations are often similar to those found in small bowel obstruction, such as dehydration and decrease in oral intake, and there may be electrolytes imbalance and acute kidney injury with raised creatinine. Abdominal X-ray findings are non-specific. CECT is a useful tool for preoperative diagnosis of abdominal cocoon [9, 10]. The imaging features are, however, not pathognomonic. CT findings of a membrane enveloping loops of small bowel were seen in some paraduodenal hernias, abdominal cocoon, and in peritoneal encapsulation. However, the clinical and pathological features of these entities are different.

Diagnostic laparoscopy is generally confirmatory and rules out other causes [11, 12]; however, laparoscopy is not helpful in management of this condition. Differential diagnosis includes peritoneal encapsulation, which was described as a developmental anomaly where the whole of the small bowel is encased in a thin accessory membrane. The clinical symptoms of this condition differ from those of abdominal cocoon syndrome, in that the patients are mostly asymptomatic and the findings are incidental and late in life.

Treatment, as in this case, is excision of membrane and releasing loops of bowel [7, 13]. Bowel resection is generally not required. However, there is scarce mention in the literature of patients who relapse with symptoms after excision of membrane. As in our case, a short course of steroids may be helpful in relapsing cases because of the inflammatory nature of this condition; however, evidence of use of steroids in cases of sclerosing peritonitis needs to be established.

## Conclusion

Sclerosing peritonitis is one of the rare causes of complete mechanical bowel obstruction and it should be in the differential diagnosis when no other obvious cause of bowel obstruction is found. Surgical exploration in which dense sclerosing membrane over the bowel is removed and bowel is straightened is the treatment of choice up until now. A short course of steroids is also helpful in the postoperative period.

## Data Availability

All data will be available on request, keeping anonymity of patient.
